# Population Pharmacokinetic–Pharmacodynamic Analysis of a Reserpine-Induced Myalgia Model in Rats

**DOI:** 10.3390/pharmaceutics16081101

**Published:** 2024-08-21

**Authors:** Gloria M. Alfosea-Cuadrado, Javier Zarzoso-Foj, Albert Adell, Alfonso A. Valverde-Navarro, Eva M. González-Soler, Víctor Mangas-Sanjuán, Arantxa Blasco-Serra

**Affiliations:** 1 Department of Human Anatomy and Embryology, University of Valencia, 46010 Valencia, Spain; gloria.alfosea@uv.es (G.M.A.-C.); alfonso.a.valverde@uv.es (A.A.V.-N.); arantxa.blasco@uv.es (A.B.-S.); 2 Department of Pharmacy and Pharmaceutical Technology and Parasitology, University of Valencia, 46100 Valencia, Spain; javier.zarzoso@uv.es; 3 Interuniversity Research Institute for Molecular Recognition and Technological Development, Polytechnic University of Valencia, University of Valencia, 46100 Valencia, Spain; 4 Systems Neurobiology, Institute of Biomedicine and Biotechnology of Cantabria (IBBTEC), Spanish National Research Council (CSIC), 39011 Santander, Spain; albert.adell@unican.es; 5 Biomedical Research Networking Centre for Mental Health (CIBERSAM), 39011 Santander, Spain

**Keywords:** reserpine, pharmacokinetic, pharmacodynamic, fibromyalgia

## Abstract

(1) Background: Fibromyalgia syndrome (FMS) is a chronic pain condition with widespread pain and multiple comorbidities, for which conventional therapies offer limited benefits. The reserpine-induced myalgia (RIM) model is an efficient animal model of FMS in rodents. This study aimed to develop a pharmacokinetic–pharmacodynamic (PK–PD) model of reserpine in rats, linking to its impact on monoamines (MAs). (2) Methods: Reserpine was administered daily for three consecutive days at dose levels of 0.1, 0.5, and 1 mg/kg. A total of 120 rats were included, and 120 PK and 828 PD observations were collected from 48 to 96 h after the first dose of reserpine. Non-linear mixed-effect data analysis was applied for structural PK–PD model definition, variability characterization, and covariate analysis. (3) Results: A one-compartment model best described reserpine in rats (V = 1.3 mL/kg and CL = 4.5 × 10^−1^ mL/h/kg). A precursor-pool PK–PD model (k_in_ = 6.1 × 10^−3^ mg/h, k_p_ = 8.6 × 10^−4^ h^−1^ and k_out_ = 2.7 × 10^−2^ h^−1^) with a parallel transit chain (k_0_ = 1.9 × 10^−1^ h^−1^) characterized the longitudinal levels of MA in the prefrontal cortex, spinal cord, and amygdala in rats. Reserpine stimulates the degradation of MA from the pool compartment (Slope_1_ = 1.1 × 10^−1^ h) and the elimination of MA (Slope_2_ = 1.25 h) through the transit chain. Regarding the reference dose (1 mg/kg) of the RIM model, the administration of 4 mg/kg would lead to a mean reduction of 65% (C_max_), 80% (C_min_), and 70% (AUC) of MA across the brain regions tested. (4) Conclusions: Regional brain variations in neurotransmitter depletion were identified, particularly in the amygdala, offering insights for therapeutic strategies and biomarker identification in FMS research.

## 1. Introduction

Fibromyalgia syndrome (FMS) is a chronic pain condition characterized by widespread musculoskeletal pain and a broad spectrum of comorbidities, which include chronic fatigue, sleep disturbances, mood alterations, cognitive impairment, and other functional symptoms [1,2]. Traditionally, pain has been the main focus of FMS treatments. Nevertheless, depressive-like symptoms, sleep disturbances, and cognitive alterations also seriously affect the individual’s quality of life, sometimes even disturbing not only for patients but also for their surroundings [2]. However, conventional therapies produce limited benefits. Even today, there is a lack of consensus regarding FMS diagnostic and classification criteria and etiopathogenesis. Various hypotheses have suggested that this syndrome involves genetic predisposition, immune system involvement, neurotransmitter dysregulation, and central sensitization [1,2,3,4]. However, the lack of knowledge of the etiopathogenesis and the mechanisms underlying FMS hinders their preclinical evaluation. Despite this, numerous animal models of FMS have been developed in recent years, and the reserpine-induced myalgia model (RIM) is the one that has managed to reproduce fibromyalgia-like symptoms better [5]. RIM was developed by Nagakura et al. in 2009 [6] since monoamines (MAs), specifically serotonin (5-HT), norepinephrine (NE), and dopamine (DA), regulate a large part of the altered processes in FMS. MAs are involved in processing pain and emotions, as well as in regulating sleep, wakefulness, and cognitive functions [7]. Furthermore, there is evidence of a decrease in MAs and their main metabolites in the CSF of people with FMS [8,9,10]. Also, antidepressant drugs that increase MA levels have shown effectiveness in symptom relief in FMS patients [11,12].

Reserpine was a first-line antihypertensive drug, but it is rarely used nowadays due to its pro-depressive side effects and the emergence of safer drugs [13]. This depressive-like symptoms can be explained by an MA depletion in neurons, mainly 5-HT, NE, and DA. Reserpine inhibits the uptake of cytosolic MAs into storage vesicles through a blockade of the vesicular monoamine transporter-2 (VMAT-2). Thus, reserpine action leads to an MA exit stoppage from the presynaptic neuron, ending up in a reduced transmission of the nervous signal in the postsynaptic neuron [13,14,15], causing a temporary decrease in MA levels. Due to its mechanism of action, the RIM model was developed at a preclinical level [5], which has been used to evaluate therapeutic alternatives in FMS [16,17,18,19,20,21].

Pharmacometrics represents an essential strategy to quantitatively characterize longitudinal pharmacokinetic (PK) and pharmacodynamic (PD) relationships with drug exposure and effect variability by integrating large, complex, and heterogeneous information, allowing a more efficient and optimal model-informed drug discovery and development process. This method allows working with sparse sampling designs with few data points per subject [22]. Therefore, this strategy perfectly contributes to a more efficient and informed drug discovery and development process together with the “3 Rs” principle (replacement, reduction, refinement), which has been highly endorsed by research and regulatory authorities in recent years [23,24,25]. They provide more accurate efficacy and safety estimates by differentiating disease changes over time from changes caused by the treatment [26,27]. Recently, disease models have been published [28,29,30,31,32,33,34], but a quantitative framework able to characterize and explore disease dynamics for FMS is still lacking. Therefore, the aims of this study were (i) to develop a population pharmacokinetic model able to characterize the time course of reserpine in rats, and (ii) to establish a population PK–PD model in rats by linking the impact of reserpine on dopamine, norepinephrine, and serotonin longitudinally in different brain regions in order to quantitatively characterize the RIM model.

## 2. Materials and Methods

### 2.1. Experimental Design and Analysis

A total of 120 male Sprague Dawley rats (Envigo RMS B.V., NM Horst, Limburg, The Netherlands) were used, which weighed in between 300 and 450 g. Animals were housed in the Central Research Unit at the University of Valencia (Spain) with a controlled cycle of 12 h light–12 h darkness at constant temperature (22 ± 2 °C) and humidity (55 ± 10%), and the air was filtered through HEPA filters and renewed more than 15 times/hour. Water and food were provided ad libitum. All the experimental protocols followed the Animal Care Guidelines of the European Communities Council Directive (2010/63/EU), Royal Decree 53/2013, and were approved by the Ethics Committee of the University of Valencia prior to performing the experiments (procedure A1546594024579). Animals were randomly assigned to experimental conditions.

Reserpine (Sigma-Aldrich, St. Louis, MI, USA) was administered once daily for three days at three dose levels: 0.1, 0.5, and 1 mg/kg. For each dose, six euthanasia times were established at the third dose: pre-dose and 30 min, 2, 4, 24, and 48 h after administration (Figure S1). Rats were anesthetized (isoflurane + O2) and euthanized by the guillotine technique.

Plasma samples were obtained immediately after decapitation, stored in heparinized vials, centrifuged, and stored at −80 °C until processing. For nervous tissue extraction, after decapitation, the brain (medial prefrontal cortex (PFC), amygdala (AMY) nuclei), and the lumbar portion of the spinal cord (SC) were extracted. Nervous tissue was stored at −80 °C until its processing.

#### 2.1.1. Plasma Reserpine Quantification

Plasma samples of reserpine were determined by liquid chromatography–mass spectrometry (LC–MS). The method was validated in terms of linearity, precision, accuracy, limit of detection and quantification, specificity, interval, and robustness. The analytical quantification of reserpine in plasma samples was conducted using an Acquity^®^ TQD mass spectrometer (Waters) under meticulously optimized conditions. Liquid chromatography (LC) separation was performed using a C18 BEH column (2.1 × 100 mm, 1.7 μm) maintained at 35 °C, with a flow rate of 0.3 mL/min in an isocratic mode. The mobile phase consisted of water with 0.5% formic acid and acetonitrile, with a gradient shift from 10% to 90% acetonitrile over a 3 min period. The injection volume was set at 5 µL. Mass spectrometry (MS) conditions included a capillary voltage of 3 KV, skimmer voltage of 5 V, and an RF lens voltage of 0.3 V. The source temperature was maintained at 120 °C, with a desolvation temperature of 350 °C, a cone gas flow rate of 25 l/h, and a desolvation gas flow rate of 650 l/h. Data acquisition was performed in multiple reaction monitoring (MRM) mode for both positive and negative ionization, ensuring precise detection of reserpine with transitions at m/z 609.2 to 192.03 and 609.2 to 395.7. The lower limit of quantification of reserpine was 0.1 μg/mL.

#### 2.1.2. Brain Monoamine Quantification

Brain tissue was homogenized in 10 volumes (*w*/*v*) of ice-cold 0.4 M perchloric acid containing 5.3 mM sodium metabisulfite, 0.27 mM EDTA and 8.3 mM L-cysteine and centrifuged for 30 min at 14,000 RPM with an Eppendorf 5430R centrifuge. Each aliquot of each supernatant was then filtered through 0.45 µm-pore Millex-HV (Merck Life Science S.L.U., Madrid, Spain) filters and assayed by HPLC using a DECADE Elite electrochemical detector. Noradrenaline, dopamine and 5-HT were determined using an Alexys Analyzer at 0.46 V (Antec Scientific, Zoeterwoude, Netherlands) following the manufacturer’s methods. Briefly, monoamines were assayed using an Acquity UPLC BEH C18 1.7 µm (1.0 × 100 mm) column (Waters Cromatografía, S.A., Cerdanyola del Vallès, Barcelona, Spain). The composition of the mobile phase was 100 mM phosphoric acid, 100 mM citric acid, 0.1 mM EDTA (adjusted at pH 6.0 with sodium hydroxide solution), 980 mg/L octane-1-sulfonic acid sodium salt, and 7% acetonitrile, and was delivered at 0.075 mL/min. The temperature of the detector was set to 42 °C. The lower limits of detection and quantification fluctuated between 1 and 5 pmol/mL. Data acquisition and calculation were carried out by Clarity chromatography software of Data Apex (Prague, Czech Republic).

### 2.2. Data Analysis

The model building, model evaluation, and simulation-based analyses were performed using non-linear mixed-effect (NLME) analysis, incorporating fixed- and random-effect parameters in Monolix software (v2024R1). RStudio software (v2023.12.1) and R**^®^** 4.2.1 (R Foundation for Statistical Computing, Vienna, Austria) were used for graphical evaluation. Population parameters were estimated using the estimation method of SAEM (stochastic approximation expectation maximization). The inter-animal variability (IAV) was modeled exponentially (Equation (1)), the distribution of which is centered on zero, and the Ω symbol represents its variance:

(1) $$P_{i}=TVP\times e^{\eta }$$

where *P_i_* represents the value of the individual parameter, *TVP* the value of the typical population parameter, and *η* the inter-animal deviation, acquired from the distribution variance (Ω). Residual variability was estimated using a proportional model, described by the following equation:

(2) $$Y=IPRED\times \left(1+\varepsilon \right)$$

where *Y* represents the experimental observations from the dataset, *IPRED* represents the individual predicted observation at time t, and *ε* the single random error effect (with mean 0 and variance σ) [35]. The significance of the non-diagonal elements of the Ω variance–covariance matrix and subject-specific residual unexplained variability were also evaluated.

The PK and PK–PD model selection was conducted through a combination of statistical, numerical, and graphical techniques. Analysis of the objective function value (OFV), which approximates to −2xlog (likelihood) for nested models, and the Bayesian information criterion (BIC) for non-nested models were used. Final parameter estimates and their relative standard error (RSE) were obtained via the Fisher information matrix, which was estimated with a stochastic approximation using a Markov chain Monte Carlo algorithm [36].

Model evaluation of the final PK and PK–PD models was performed through prediction-corrected visual predictive checking (pc-VPC) with 1000 datasets obtained by Monte Carlo simulation using the final parameter estimates for both fixed and random effects [37]. Each simulated dataset had study design features (covariates, dosing times, and PK sampling times) identical to those in the analysis dataset. For each simulated dataset, the 2.5th, 50th, and 97.5th percentiles of the simulated concentrations in each bin were calculated. Then, the 95% prediction intervals of these percentiles were calculated and displayed graphically, together with corresponding percentiles computed from raw data. In addition, goodness-of-fit plots to assess the performance of the final PK and PK–PD model were built.

#### 2.2.1. Population Pharmacokinetic Model

Reserpine plasma levels were described with PK compartmental models parameterized in apparent volumes of distribution, as well as first-order distribution and elimination clearances. Non-linear processes on distribution and elimination through Michaelis-Menten equations were also evaluated.

#### 2.2.2. Population Pharmacokinetic–Pharmacodynamic Model

The time course of MAs after reserpine administration was determined through a compartmental approach, evaluating linear and non-linear processes under the principle of parsimony [38,39]. Different structures (turnover response, precursor-pool, and transduction models) were combined to elaborate a PK–PD structure able to characterize the longitudinal MA levels across different brain regions.

Covariates were tested on the PK–PD model, which consisted of the brain area and the type of MA. A comparison of final parameter estimates was conducted after the addition of each covariate vs. the base model. The covariate was retained if a statistically significant reduction in the OFV was observed (*p*-value < 0.05). This step was repeated until the inclusion of other covariates was not statistically significant.

### 2.3. Simulation-Based Analysis

Monte Carlo simulations (*n* = 10,000) were conducted assuming a log-normal distribution of PK–PD parameters to reproduce different FMS disease statuses. Once-daily (QD) dosage tested (0.1, 0.5, and 1 mg/kg) and untested (2 and 4 mg/kg) regimens were considered. Several PD outcomes were evaluated to internally validate the PK–PD framework and to understand the rate (C_max_ and C_min_) and extent (area under the curve: AUC) of disease status achieved.

## 3. Results

### 3.1. Dataset and Data Exploration

For PK model development, 120 samples (i.e.*,* one sample per animal) were available. Experimental MA (5-HT, DA, and NE) observations (*n* = 828) after reserpine administration were obtained in AMY, PFC, and SC. PK and PD samples were collected from 48 to 96 h after the first dose was administered. Table 1 summarizes the number of samples across the dose levels evaluated and brain regions, while Figure S2 shows graphical representation of the experimental PK and PD samples across different reserpine doses. Individual longitudinal PK and PD profiles were created with a pre-established combination of samples from different animals.

### 3.2. Data Analysis

#### 3.2.1. Population PK Model

A one-compartment model with double extravascular absorption and linear elimination was selected based on the OFV and AIC criteria. The absorption was modeled with a simultaneous first-order rate constant (ka_1_= 19.14 h*^−^*^1^/kg) and a zero-order rate process (ka_2_ = 44.69 mg/h/kg). A proportion of 96% (F1) of the administered dose was absorbed through the linear process. Linear disposition processes (V and CL) were assumed for reserpine in the central compartment.

#### 3.2.2. Population PK–PD Model

**Figure 1:**
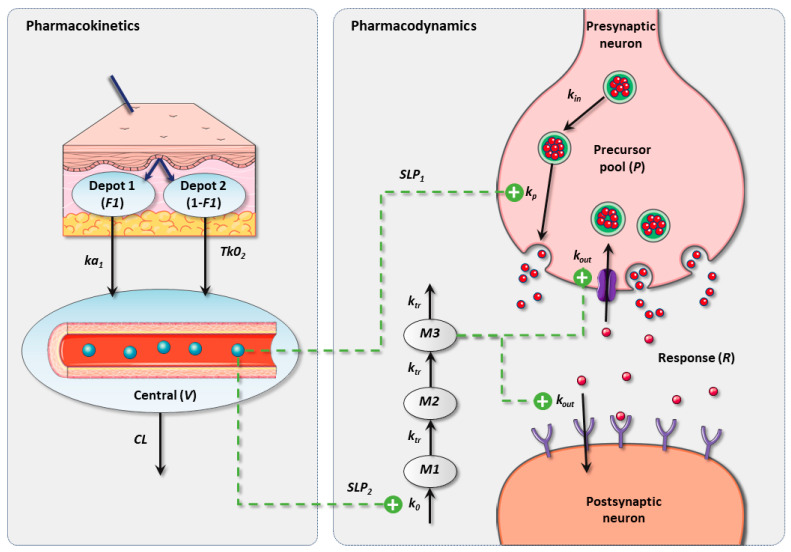
Schematic representation of the final PK–PD model in rats. PK: pharmacokinetic; PD: pharmacodynamic; V: apparent volume of distribution; CL: elimination clearance; ka_1_: first-order absorption rate constant; Tk0_2_: duration of zero-order absorption; F1: fraction absorbed by means of ka_1_; k_in_: precursor production rate constant; k_p_: response production rate constant; k_out_: response degradation rate constant; k_0_: transit response production rate constant; k_tr_: transit response rate constant.

The final PK–PD framework included a precursor-dependent model, whose structure was defined by Sharma et al. [40] and adapted to best fit the study data. Figure 1 illustrates a schematic representation of the population PK–PD model for reserpine and MA.

**Figure 2:**
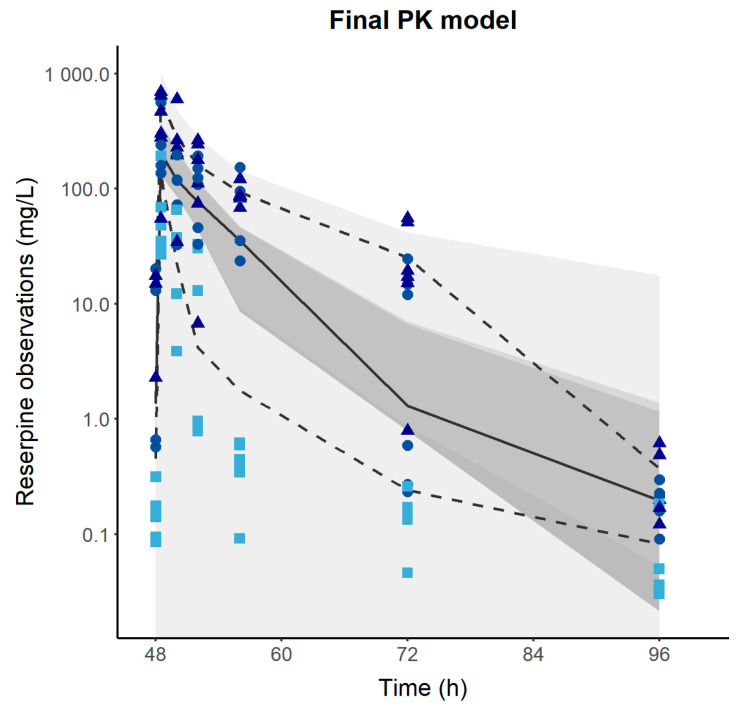

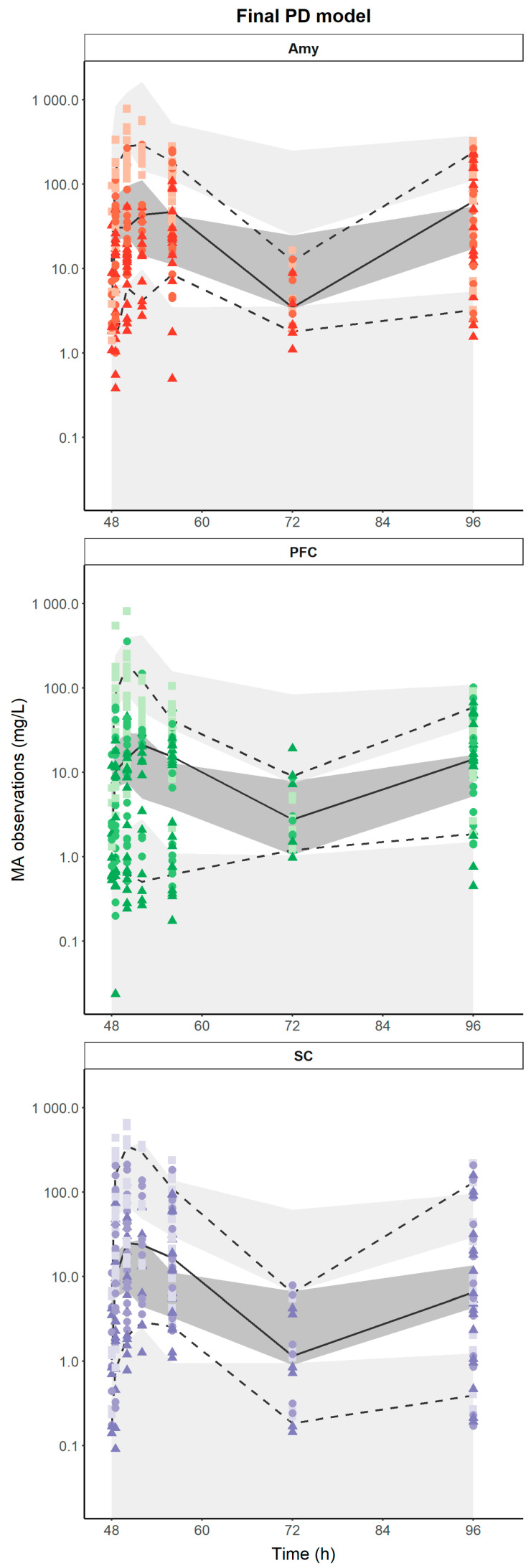
Prediction-corrected visual predictive check of the final population pharmacokinetic/pharmacodynamic model. Dashed and solid lines represent the experimental 2.5th, 50th, and 97.5th percentiles. Gray-shaded areas represent the 95% prediction interval of the 2.5th, 50th, and 97.5th percentiles. Colored dots represent the reserpine or MA observations. Circles, triangles, and squares represent the 0.1, 0.5, and 1 mg/kg doses.

Table 2 lists the parameters of the final PK–PD model, which involves a precursor pool (P) produced at a zero-order process (k_in_ = 6.1 *×* 10*^−^*^3^ mg/h), and the response (R) is mediated and eliminated through first-order processes (k_p_ = 8.6 *×* 10*^−^*^4^ h*^−^*^1^ and k_out_ = 2.7 *×* 10*^−^*^2^ h*^−^*^1^). Parallel to this process, a three-transit compartment chain (M1, M2, M3) is incorporated, governed by a zero-order production rate constant (k_0_ = 1.9 *×* 10*^−^*^1^ h*^−^*^1^) and assuming the initial condition M1_0_ = M2_0_ = M3_0_ = 1. The amount of reserpine in the central compartment stimulates the transit from P to R (SLP_1_ = 1.1 *×* 10*^−^*^1^ h) and the degradation of R through a linear drug effect model (SLP_2_ = 1.25 h). The covariate analysis identified the brain regions (PFC, SC, and AMY) as statistically significant covariates on k_in_, which could be a consequence of different baseline levels of MA across the brain regions. Low-to-moderate IAV was estimated for most of the PK–PD parameters, except for ka_1_ (209%), F1 (170%), and SLP1 (358%). Moderate RUV (residual unexplained variability) was obtained for the PK (56%) and PD (71%) observations, which was expected based on the study design characteristics of the longitudinal profiles. Figure 2 depicts the final model evaluation (pc-VPC) of the population PK–PD model, suggesting that the PK–PD framework is capable of characterizing both the median tendency and the dispersion of the data. Goodness-of-fit plots showed an acceptable degree of performance of the model in describing the experimental data (Figures S3 and S4).

### 3.3. Simulation-Based Analysis

**Figure 3:**
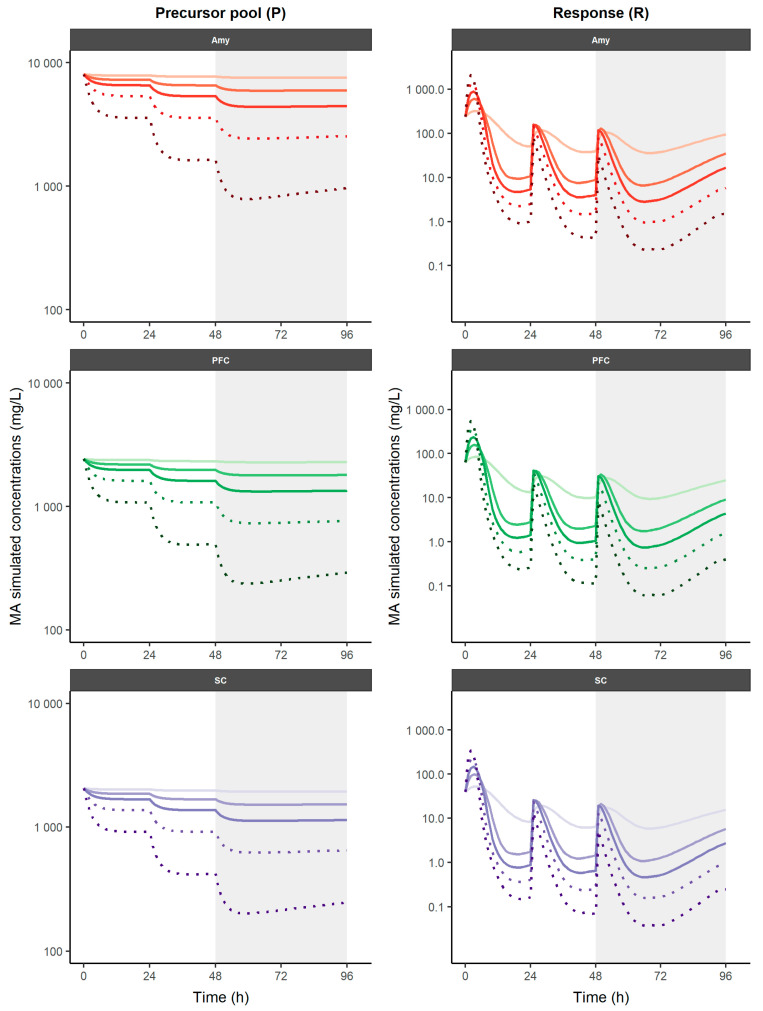
Simulation of time-course profiles on different nervous tissues obtained using PK–PD model for neurotransmitters after different reserpine dosage regimens. Experimental doses are represented as solid lines, additional simulated doses are represented as dotted lines. A gradual increase in color intensity represents the level of dose considered (0.5 to 4 mg/kg). Shaded areas represent the study period in the experimental protocol. MA: monoamine; AMY: amygdala; PFC: prefrontal cortex; SC: spinal cord.

The simulation-based analysis offered a primary internal validation by simulating experimental dosage regimens (0.1, 0.5, and 1 mg/kg QD for three consecutive days) and a posterior model application with additional doses (2 and 4 mg/kg QD for three consecutive days). The simulation profiles using the PK–PD model across all dosing strategies are presented in Figure 3. When increasing doses of repetitive reserpine administrations, higher MA depletion is achieved and C_max_ and C_min_ become steeper, while lower doses lead to higher and steadier MA concentrations.

**Figure 4:**
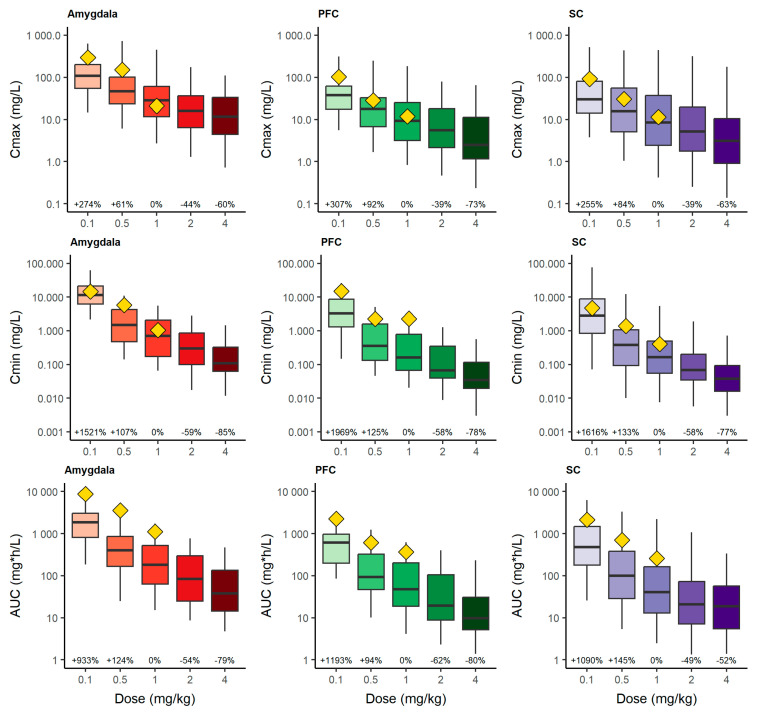
Numerical predictive check. Simulated C_max_ (**top**), C_min_ (**middle**) and pharmacodynamic AUC (**bottom**) of neurotransmitters at different simulated dosage regimens of reserpine. Evaluation was performed for the study period after the third reserpine administration (48 h) until the end of the study period (96 h). Yellow diamonds represent the experimental median values for each parameter. Percentage values represent the percentage change in the median with respect to 1 mg/kg dose. PFC: prefrontal cortex; SC: spinal cord.

The numerical predictive check for C_max_, C_min_, and AUC is displayed in Figure 4. Regarding the reference dose (1 mg/kg) of the RIM model, the administration of 2 mg/kg would provide a median reduction of 44%, 39%, and 39% (C_max_), 59%, 58%, and 58% (C_min_) and 54%, 62%, and 49% (AUC), whereas 4 mg/kg would lead to a median reduction of 60%, 73%, and 63% (C_max_), 85%, 78%, and 77% (C_min_), and 79%, 80%, and 52% (AUC) across AMY, PFC, and SC, respectively. The results show a proportional reduction between 1 and 2 mg/kg, but a less than proportional reduction between 2 and 4 mg/kg, which could be indicating a complete depletion of the precursor, affecting the prediction of more severe or advanced FMS stages.

## 4. Discussion

FMS is a disease with a lack of understanding regarding its etiopathogenesis [3], but several MAs have been demonstrated to be implicated in the underlying mechanisms [1]. Nevertheless, no mathematical approaches account for these MA alterations in FMS, revealing a scientific gap of knowledge. The current population PK–PD model of RIM, the most accurate animal model of FMS, characterizes the reserpine-induced mechanism in CNS and improves the evaluation of pharmacological therapies under different disease status conditions.

After repetitive reserpine administration, the longitudinal changes in MA levels showed an initial and rapid increase, with a subsequent MA depletion to the minimum around 72 h and a recovery that slightly approached baseline levels at 96 h. The proposed PK–PD framework characterized these patterns. This is in accordance with previous studies that were related to depressive and pain-related symptoms, which showed a decrease in MA levels after repetitive reserpine administration [6,16,18,41,42].

The influence of reserpine on the longitudinal MA profiles varies across different brain regions, as demonstrated and characterized in the current work (Figure 3). The more remarkable MA synthesis of the analyzed centers is found in the AMY precursor pool. In fact, in PFC and especially in SC, it is observed that at the highest doses simulated (2 and 4 mg/kg), a depletion in the precursor pool occurs, which leads to a depletion in MA levels in the response compartment (Figure 3). Reserpine administration affects the neurotransmission of the AMY differently than the other studied areas related to pain processing, which could lead to a pattern of functional alterations in the model, like those observed in FMS. Morphometric, connectivity, and functional alterations have been found in pain processing areas in patients with FMS, especially in those related to the affective–motivational aspects of pain [43,44,45,46]. Regarding this, the AMY plays a crucial role in the affective component of pain, and the aberrant activation of the AMY in pain-related fear has been proposed as a biomarker of FMS [47].

The PK–PD relationship between reserpine and MAs was described with a linear drug effect model across the dose range (0.1–1 mg/kg) evaluated, indicating a proportional relationship between reserpine exposure and response (MAs) [34,48]. The additional dose levels tested (2 and 4 mg/kg) after three consecutive daily doses would provide reductions of about 50% and 75% in the different PD outcomes (AUC, C_0_, C_max_, and C_min_) in all brain regions in rats, which could contribute to a more individualized design of new pharmacological candidates for the different brain regions, as well as the impact of the dose on the degree of disease. Nevertheless, it must be considered that the precursor-pool model contemplates the saturation of the response due to the depletion of the levels in the pool compartment, which appears to occur at 4 mg/kg.

Despite the rapid elimination of reserpine (t_1/2_ = 2.25 h) and the recovery of baseline MA levels around 96 h, similar behavioral alterations to those described in FMS persist or appear in subsequent weeks [6,16,17,19]. For example, FMS-like sleep disturbances develop from the third week after reserpine administration [49]. Therefore, reserpine administration can trigger other pathophysiological mechanisms that may not directly relate to MA levels.

The abrupt depletion of MAs in the system can lead to plastic changes that permanently modify connectivity [50]. On the other hand, the massive accumulation and degradation by MAO (monoamine oxidase) of MAs accumulated in the cytosol of monoaminergic neurons that cannot be released by VMAT-2 blockade can lead to cytotoxicity, neuroinflammation, or cell death [51]. In this respect, glial cells could be involved. It has been described that the mechanisms that lead to chronic pain appear to be a glial and immune interaction [2,50,52]. Research on these interactions shows that glia-mediated neuroinflammation is a key mechanism underlying the maintenance of chronic pain [53,54]. Furthermore, it has recently been described that mammalian astrocytes have VMAT-2 receptors, and it is necessary to know how the administration of reserpine can affect their functioning and influence the symptoms generated [55]. Studying the neuroimmune and glial processes present in the RIM model can shed light on the pathophysiological mechanisms involved in FMS. Finally, reserpine has a sympatholytic effect [13]. Long-term consequences of NE depletion in the sympathetic terminals produced by reserpine administration could be relevant scientifically, since FMS has been considered a stress-related pathology [2,4,10] and the autonomic nervous system is altered in people with FMS [56].

The study and the development of the present PK–PD model had a few limitations. Due to the study design conditions, longitudinal profiles were constructed from different rats, leading to an IAV and residual error increase. Moreover, no covariates (body weight, age, breed, or sex) were statistically significantly different on PK or PD parameters in order to explain the large IAV, except the brain region on k_in_, due to the low variation of these covariates among animals. Additionally, although the proposed model explains the longitudinal pattern of MAs, the methodology of dissection and homogenization of areas of interest for counting MAs does not allow us to verify whether the measured MAs are found in the presynaptic or postsynaptic neuron or in the synaptic cleft. In vivo studies would be necessary to corroborate the model. Finally, further studies are necessary to assess whether this model can be translated into clinical conditions.

## 5. Conclusions

In conclusion, this study successfully developed and validated a pharmacokinetic (PK) coupled with a pharmacodynamic (PD) model for characterizing the rapid depletion of a precursor pool with a delayed effect on the degradation of MAs in different regions of the rat brain. The model was evaluated after three daily administrations of 0.1, 0.5, and 1 mg/kg of reserpine in rats. The evaluation of pharmacodynamic outcomes revealed that the concentration of MAs in the different brain regions changed proportionally across the dose levels evaluated. However, the impact of reserpine on the longitudinal MA profiles varied across different brain regions, with a greater MA synthesis from the AMY precursor pool. The developed PK–PD model can be a powerful tool for correlating MA levels with behavioral and biochemical results obtained with the RIM model. This may be useful in searching for biomarkers in FMS and translating the results of preclinical studies to human research. Future studies should analyze the pathophysiological mechanisms in the nervous system due to reserpine administration and correlate them with MA levels using the proposed PK–PD model. Additionally, they should also investigate possible neuroinflammation processes and glial alterations that may lead to the FMS-like symptoms present in the RIM model.

## Figures and Tables

**Table 1:** Number of samples obtained per dose group (PK model) and per NT and nervous tissue (PD model).

Dose Group	Number of PK Samples	Number of PD Samples								
		5-HT			DA			NE		
		AMY	PFC	SC	AMY	PFC	SC	AMY	PFC	SC
0.1 mg/kg	39	31	31	31	30	28	31	29	31	31
0.5 mg/kg	40	30	31	31	31	31	31	28	31	31
1.0 mg/kg	41	31	31	31	31	32	31	31	31	31
**Total**	**120**	**278**			**276**			**274**		

PK: pharmacokinetic; PD: pharmacodynamic; 5-HT: serotonin; DA: dopamine; NE: norepinephrine; AMY: amygdala; PFC: prefrontal cortex; SC: spinal cord.

**Table 2:** Final population pharmacokinetic–pharmacodynamic parameters of reserpine and neurotransmitters in rats.

Parameters	Population PKPD Model Estimates				Bootstrap Results	
	Fixed Effects		Inter-Animal Variability		Fixed Effects	Inter-Animal Variability
	Value	RSE (%)	Value	RSE (%)	Median Value (2.5th–97.5th Percentiles)	Median Value (2.5th–97.5th Percentiles)
ka_1_ (h^−1^/kg)	19.14 FIX	-	226	28	19.14 FIX	221 (135–379)
ka_2_ (mg/h/kg)	45.43	12	32	-	44.78 (36.06–57.22)	30
F1	0.95	3	179	22	0.94 (0.87–0.99)	177 (119–270)
V (mL/kg)	1.3	21	59	30	1.2 (0.84–1.9)	53 (34–102)
CL (mL/h/kg)	4.5 × 10^−1^	11	37	25	4.7 × 10^−1^ (3.6 × 10^−1^–5.6 × 10^−1^)	35 (23–60)
k_in_ (mg/h) AMY	6.97	18	97	9	7.04	96 (81–116)
k_in_ (mg/h) PFC	2.10	18			2.16	
k_in_ (mg/h) SC	1.78	19			1.76	
k_p_ (h^−1^)	8.6 × 10^−4^	14	29	37	8.4 × 10^−4^ (6.6 × 10^−4^–1.1 × 10^−3^)	25 (15–57)
k_out_ (h^−1^)	2.7 × 10^−2^	11	22	24	2.6 × 10^−2^ (2.2 × 10^−2^–3.4 × 10^−2^)	25 (14–35)
SLP_1_ (h)	1.1 × 10^−1^	47	358	11	1.3 × 10^−1^ (4.9 × 10^−2^–2.5 × 10^−1^)	344 (291–440)
k_0_ (h^−1^)	1.9 × 10^−1^	6	9	67	2.1 × 10^−1^ (1.7 × 10^−1^–2.1 × 10^−1^)	10 (3–25)
SLP_2_ (h)	1.25	20	74	18	1.23 (0.85–1.83)	72 (53–104)
**Residual unexplained variability**						
PK (%)	54	9			56 (45–65)	
PD (%)	71	9			72 (65–77)	

RSE (%): residual standard error expressed as percentage; ka_1_: first-order absorption rate constant; ka_2_: duration of zero-order absorption; F1: fraction absorbed by means of ka_1_; V: apparent volume of distribution; CL: elimination clearance; k_in_: precursor production rate constant; k_p_: response production rate constant; k_out_: response degradation rate constant; SLP_1_: slope relating reserpine levels with k_p_ stimulation; k_0_: transit response production rate constant; SLP_2_: slope relating transit compartments with k_out_ stimulation; PK: pharmacokinetic; PD: pharmacodynamic.

## Data Availability

The data that support the findings of this study are openly available on Zenodo at http://doi.org/10.5281/zenodo.11206173 (accessed on 16 May 2024).

## Supplementary Materials

The following supporting information can be downloaded at https://www.mdpi.com/article/10.3390/pharmaceutics16081101/s1. Figure S1: Reserpine administration and sampling schedule scheme; Figure S2: Individual longitudinal PK and PD profiles obtained from experimental data across different reserpine doses; Figure S3. Goodness-of-fit plots of the final population pharmacokinetic model of reserpine in rats; Figure S4. Goodness-of-fit plots of the final population pharmacokinetic-pharmacodynamic model of reserpine in rats.
