# Supplementary material for: Population Pharmacokinetic–Pharmacodynamic Analysis of a Reserpine-Induced Myalgia Model in Rats
Source: Pharmaceutics. 2024 Aug 21;16(8):1101. doi: 10.3390/pharmaceutics16081101 (PMC11359992; doi:10.3390/pharmaceutics16081101)
Supplement: Supplementary file 1 [file pharmaceutics-16-01101-s001.zip › pharmaceutics-3140028-supplementary.pdf]

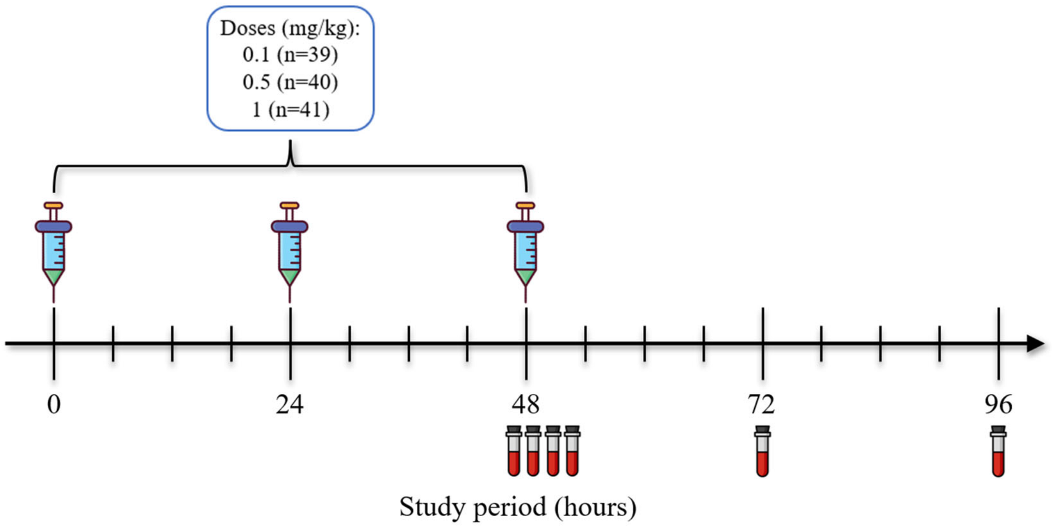

Figure S1. Reserpine administration and sampling schedule scheme. Syringes represent reserpine administrations, and tubes represent the sacrifice and sampling time points. From each individual, 1 plasma and 3 nervous tissue samples (amygdala, PFC, and SC) were obtained.

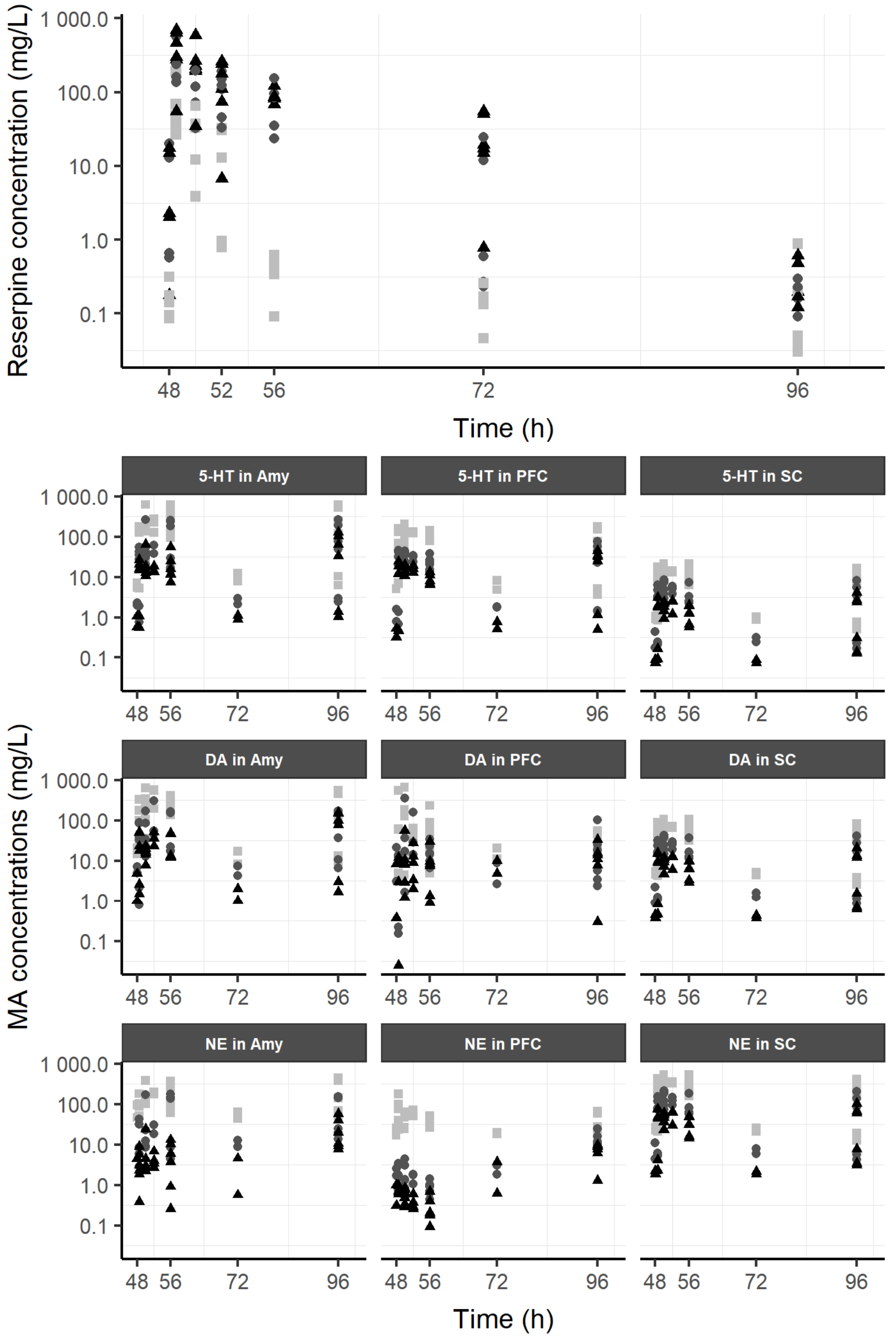

Figure S2. Individual longitudinal PK and PD profiles obtained from experimental data across different reserpine doses (squares, circles, and triangles for 0.1, 0.5 and 1 mg/kg, respectively. Profiles were created with a pre-established combination of samples from different individuals; MA: monoamines.

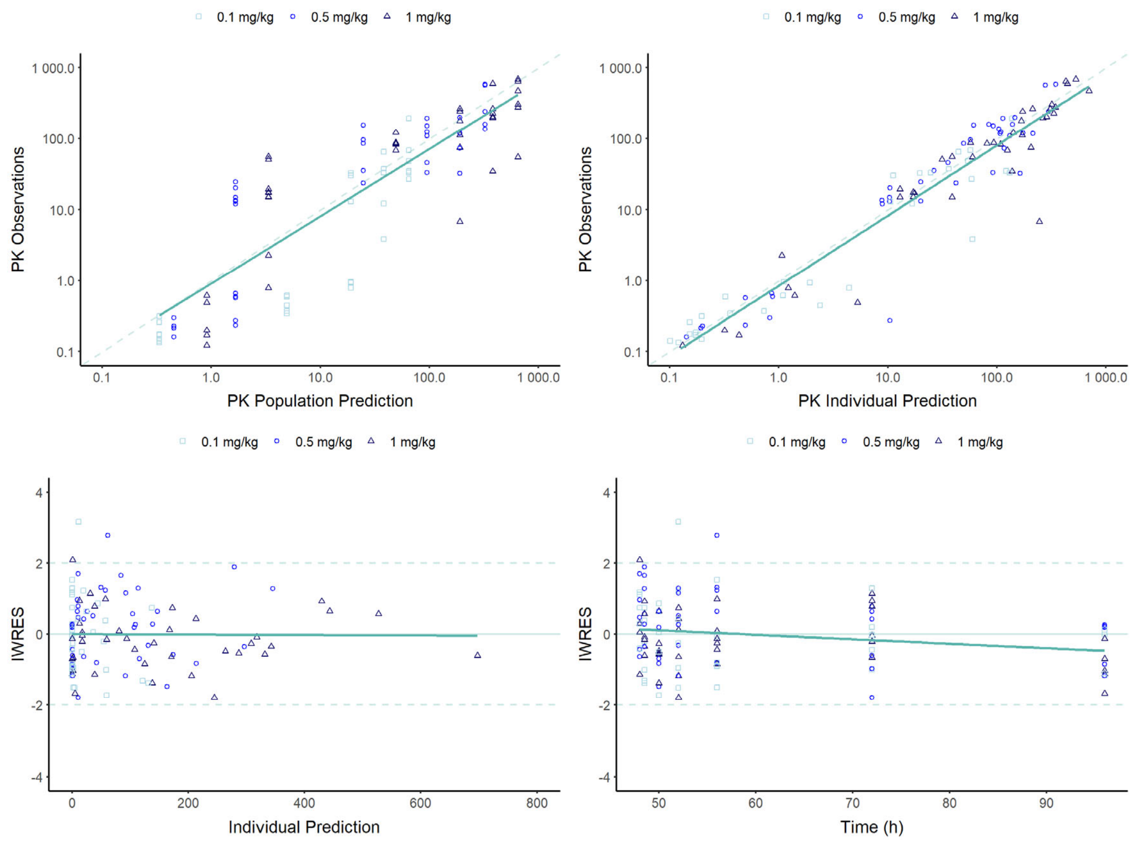

Figure S3. Goodness-of-fit plots of the final population pharmacokinetic model of reserpine in rats. The blue solid line represents the non-linear regression and the blue dotted line represents the line of identity or the ±2 units. IWRES: individual weighted residuals.

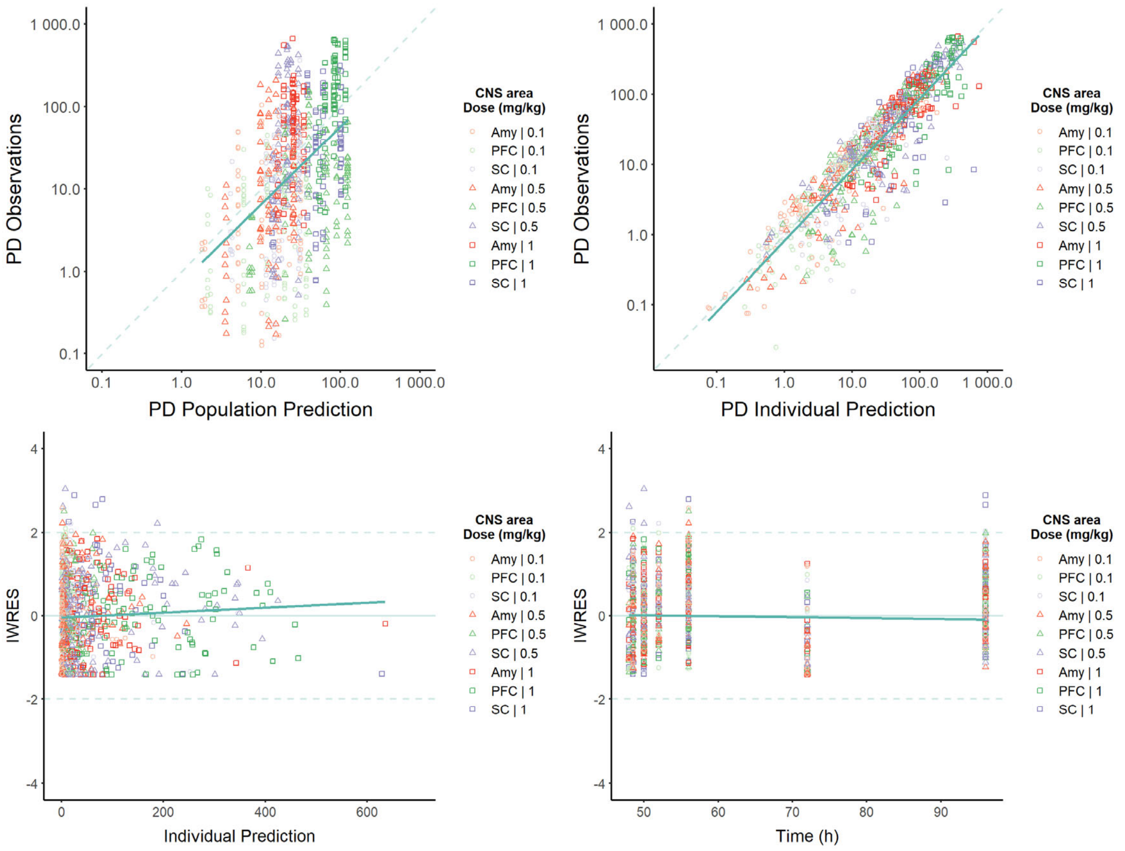

Figure S4. Goodness-of-fit plots of the final population pharmacokinetic-pharmacodynamic model of reserpine in rats. The blue solid line represents the non-linear regression and the blue dotted line represents the line of identity or the ±2 units. IWRES: individual weighted residuals.Amy: amygdala; PFC: prefrontal cortex; SC: spinal cord.
